# Jump around: transposons in and out of the laboratory

**DOI:** 10.12688/f1000research.21018.1

**Published:** 2020-02-24

**Authors:** Anuj Kumar

**Affiliations:** 1 Department of Molecular, Cellular, and Developmental Biology, University of Michigan, Ann Arbor, MI, USA; 2 Program in Cellular and Molecular Biology, University of Michigan, Ann Arbor, MI, USA

**Keywords:** transposable elements, transposon mutagenesis, insertion library, phenotypic screening, genomics, CRISPR-Cas

## Abstract

Since Barbara McClintock’s groundbreaking discovery of mobile DNA sequences some 70 years ago, transposable elements have come to be recognized as important mutagenic agents impacting genome composition, genome evolution, and human health. Transposable elements are a major constituent of prokaryotic and eukaryotic genomes, and the transposition mechanisms enabling transposon proliferation over evolutionary time remain engaging topics for study, suggesting complex interactions with the host, both antagonistic and mutualistic. The impact of transposition is profound, as over 100 human heritable diseases have been attributed to transposon insertions. Transposition can be highly mutagenic, perturbing genome integrity and gene expression in a wide range of organisms. This mutagenic potential has been exploited in the laboratory, where transposons have long been utilized for phenotypic screening and the generation of defined mutant libraries. More recently, barcoding applications and methods for RNA-directed transposition are being used towards new phenotypic screens and studies relevant for gene therapy. Thus, transposable elements are significant in affecting biology both *in vivo* and in the laboratory, and this review will survey advances in understanding the biological role of transposons and relevant laboratory applications of these powerful molecular tools.

## Introduction

Transposons are mobile repetitive genetic elements that are widespread throughout prokaryotic and eukaryotic genomes, considerably impacting many facets of biology, including genome evolution, genome composition, and human health ^1– 5^. The spread of multiresistant bacterial strains is an increasing healthcare problem worldwide, and the acquisition of pre-existing antibiotic resistance determinants is commonly achieved through the actions of mobile genetic elements, notably including transposons (reviewed in 6). Somatic transposition is increasingly being recognized for its biological and health-related significance. Postzygotic retrotransposition can occur in healthy and diseased cortical neurons and non-brain tissue ^7^, and somatic retrotransposon insertions have been reported in cancer patients, with high retrotransposition rates in tumors associated with high rates of genomic rearrangement and somatic mutation ^8, 9^. Transposon mobilization is highly mutagenic, as the insertion of a transposable DNA sequence is likely to perturb native gene expression and/or function at the locus of insertion ^10, 11^. The consequences of transposon insertion include open reading frame disruption, the alteration of promoter sequence, perturbed splicing and transcriptional termination, and epigenetic effects impacting nearby sequences. Consequently, organisms have established extensive mechanisms to combat transposition ^12– 16^. Despite these genome defense mechanisms, transposable elements constitute approximately 46% of the human genome ^17^, and the interplay between transposition and host mechanisms is an ongoing area of study. This review will highlight current thought regarding this interplay, which involves commensal or mutualistic strategies. Recent studies have further shed light on the impact of transposition on the expression of genes at the locus of insertion, and these findings will be reviewed here. Transposons have been and remain a relevant tool in the laboratory, and this review will summarize several advances in the application of transposable elements for mutagenesis and molecular biology applications. Collectively, this review is designed to update our understanding of transposons in the context of evolutionary biology, molecular genetics, and biotechnology.

## Transposable elements and host interactions

Transposable elements are predominantly viewed as “selfish” DNA elements, replicating to great numbers in many genomes ^18^. Although a full summary of transposon classes and structures is beyond the scope of this text, transposable elements have been divided into two large classes according to the employed method of transposition. Class 1 elements, or retrotransposons, transpose through an RNA intermediate by the action of reverse transcriptase. These elements can be subdivided into classes based on the presence or absence of long terminal repeats (LTRs) and further by the autonomy of the element—its ability to encode the necessary proteins for transposition. Long INterspersed Elements (LINEs) are autonomous non-LTR retrotransposons, and Short INterspersed Elements (SINEs) are non-autonomous and highly abundant non-LTR retrotransposons. Human *Alu* elements, each approximately 300 base pairs (bp) in length, are among the most abundant SINEs observed in any organism; over one million copies of *Alu* elements are dispersed throughout the human genome ^17^. Although immobile, endogenous retroviruses (ERVs) are a type of abundant retroelement, with human ERVs (HERVs) accounting for 8% of the human genome ^17^. HERVs have shaped the evolution of the human genome, regulatory networks, and innate immune responses ^19^. The residual expression capacity of many HERVs can regulate genes and influence host immunity ^20, 21^. Class 2 elements, or DNA transposons, transpose through a mechanism involving a DNA intermediate. DNA transposons are estimated to constitute over 3% of the human genome, encompassing at least 125 different families exhibiting a respective copy number of 100 or more ^22^.

Transposon insertions are heritable and may spread vertically within a population and horizontally within species ^23, 24^. Natural selection and genetic drift are important determinants of the evolutionary fate of transposon insertions, with most extant insertions being neutral or only mildly deleterious to the host ^25^. Upon initial transposition, however, the majority of insertions are presumed to be disruptive of gene function at the insertion locus. Additionally, transposon insertions, whether deleterious or not, may promote chromosomal rearrangements by providing foci for non-allelic homologous recombination ^1, 26, 27^. Consequently, numerous mechanisms have evolved through which host organisms moderate or tolerate transposition.

A variety of regulatory mechanisms functioning at the transcriptional and post-transcriptional levels act to limit transposition. In eukaryotes other than plants, PIWI-interacting RNAs (piRNAs) are a primary and well-studied mechanism of transposon silencing ^14, 28, 29^. piRNA-mediated inhibition of transposition is reviewed in Ozata *et al.* ^30^. In animals and plants, small interfering RNAs (siRNAs) derived from transposable element loci trigger transposon silencing. Small RNAs can inhibit the transcription of neighboring genes at the site of transposon insertion through the deposition of repressive epigenetic modifications ^31^. In mouse embryonic stem cells, transposable elements are suppressed by heterochromatic histone modifications, such as H3K9me3, and are regulated by a host of epigenetic modifiers ^32^. In mammals, Kruppel-associated box (KRAB) zinc-finger proteins bind transposable element sequences, with significant impact on retrotransposons, and recruit KRAB-associated protein-1 (KAP1/TRIM28), nucleating interactions with multiple proteins that generate a repressive chromatin architecture at the transposon insertion locus ^33– 36^.

DNA methylation has been recognized as an important mechanism combating transposition. Methylation and other means by which DNA modifications regulate transposition are reviewed in Deniz *et al.* ^37^. ATP-dependent chromatin remodelers in mammals and plants recruit methylases to produce a repressive chromatin state inhibiting transposition ^16, 38, 39^. 5-methylcytosine is one of the more well-studied DNA modifications, and cytosine methylation is linked with repressed transposition ^40– 44^. In zebrafish embryos, global DNA hypomethylation caused by mutations in the DNA methyltransferase gene *dnmt1* has been associated with widespread induction of class I retrotransposons and subsequent activation of cytoplasmic DNA sensors, mimicking a viral infection ^45^. In early studies, Zhou and colleagues ^46^ found that the *Neurospora crassa* LINE-like *Tad* retrotransposon inserted in the 5'-non-coding sequence of the *am* glutamate dehydrogenase gene ** carries a *de novo* cytosine methylation signal that causes reversible methylation of *Tad* and *am* upstream sequences. This methylation inhibits *Tad* expression and transposition, and the inhibition can be relieved by treatment with the drug 5-azacytidine, which reduces cytosine methylation. In addition to 5-methylcytosine, several studies suggest a role for *N*6-methyladenine in regulating transposition ^47, 48^. *N*6-methyladenine has been identified across prokaryotes, archaea, and eukaryotes, although it is not abundant in metazoans ^49– 52^. In *Escherichia coli*, activity of the Tn *10* transposon is highly elevated in strains with decreased levels of *N6*-methyladenine from mutation of the *dam N*6-methyladenine methyltransferase ^48^. In zebrafish, *N*6-methyladenine is enriched at repetitive elements, including *LINE-1*, LTR, and DNA transposable elements ^53^.

As proposed by Barbara McClintock and subsequent researchers, transposons have evolved commensal or mutualistic strategies with host organisms, contributing to the widespread evolutionary success of transposable elements ^54^. Mutualism has been commonly observed in prokaryotes, as transposons and conjugative plasmids frequently shuttle antibiotic resistance genes ^55^. Prokaryotic mobile genetic elements may carry genes beneficial to their host, encoding secretion proteins, cation efflux pumps, copper resistance proteins, and proteins in restriction modification systems ^56^. Numerous catabolic genes are present on transposons, including insertion sequence composite transposons, underlying the tendency of many catabolic genes to undergo genetic rearrangements ^57^. The origin of the CRISPR (Clustered Regularly Interspaced Palindromic Repeats)–Cas system presents a striking example of mutualism between host and mobile genetic elements. Over the past 15 years, we have observed the widespread recognition of CRISPR–Cas systems as an adaptive immune response in bacteria and archaea ^58– 61^. By these well-studied systems, “spacer” sequences from phage and plasmids are inserted into CRISPR sequence arrays; resulting CRISPR transcripts are processed, such that the phage sequences are loaded onto Cas proteins for recognition of the foreign genome. The CRISPR–Cas adaptation module for the integration of foreign DNA fragments as unique spacers in the CRISPR array is proposed to have evolved from a superfamily of Cas1-encoding genetic elements that were likely mobile, termed *Casposons* (reviewed in 62, 63).

Examples of mutualism are also evident in eukaryotes. In jawed vertebrates, the Recombination Activating Gene (RAG) proteins 1 and 2 mediate the site-specific double-stranded DNA breaks necessary for V(D)J recombination and share mechanistic and structural similarities with several families of transposases ^64^. Notably, the RAG proteins are thought to have evolved from the *ProtoRAG* DNA transposon family ^65^. *ProtoRAG* was demonstrated to encode RAG1- and RAG2-like proteins that constitute an active endonuclease and transposase *in vitro* and in living cells ^65^, and structural analysis through X-ray crystallography and cryo-electron microscopy of the RAG1-like transposase *Transib* from the moth *Helicoverpa zea* has identified many mechanistic details relevant to our understanding of cut-and-paste transposition ^66^. Transposable elements may contribute to the establishment of telomere-like sequences in Drosophilid species lacking telomerase. In nearly all *Drosophila* species, telomeric repeats have been replaced with arrays of non-LTR retrotransposon sequences ^67^. In *Drosophila melanogaster*, three families of *Jockey*-like retrotransposons act cooperatively to enable their own amplification and, consequently, the maintenance of telomeric sequence ^68, 69^. Furthermore, the *G2*/ *Jockey-3* family of non-LTR retrotransposons contributes directly to the function and organization of centromeric sequences in *D. melanogaster* ^70^. Transposable elements can carry virulence genes in some fungal pathogens ^71^. In the ciliate *Oxytricha trifallax*, a family of DNA transposons are mobilized during meiosis, cooperatively contributing to the remodeling of the germline micronucleus and somatic macronucleus. RNAi-based silencing of the transposases encoded by this family of transposons impairs cell growth and causes cell death owing to aberrant micronuclear and macronuclear development ^72– 75^. Proteins derived from ERVs have been coopted repeatedly to promote cell–cell fusion, regulate the expression of genes important for human pregnancy, and modulate immune responses in the placenta ^76, 77^. Cosby *et al.* ^54^ further review host–transposon interactions towards understanding the impact of transposition on genome organization and biology.

## Transposable elements modulate gene expression

Transposons are highly abundant in genomes and can encode promoter sequences, splice sites, transcriptional terminator sequences, binding sites for multiple transcription factors, and sequences that modify chromatin conformation ^78– 80^. Consequently, transposable elements play significant roles in regulating the expression of nearby genes ^81^, and important findings elucidating the regulatory role of transposons in modulating gene expression are reviewed in Rebello *et al.* ^82^.

From an evolutionary perspective, transposable elements may have played an important role in the development of transcriptional regulatory networks, as internal promoters and binding sites for host transcription factors are evident in transposable element sequences ^83^. Transposition may have promoted the distribution of these regulatory elements, with subsequent selection resulting in the evolution of regulatory pathways ^84, 85^. Among these regulatory elements, enhancer-like epigenetic features have been identified, particularly in the LTRs of ERVs. Todd *et al.* ^86^ identified a large set of putative enhancers overlapping with ERV-encoded LTRs in mouse embryonic and trophoblast stem cells, although *in situ* evaluation of enhancer activity indicates that the majority of these elements do not exhibit enhancer function. Work in humans ^87, 88^ indicates that primate-specific LTRs encoding putative enhancers impact gene transcription *in situ* to a greater degree than do the elements observed in mouse stem cells, highlighting the need for further consideration of these sequences. Tellier and Chalmers ^89^ identified a broad impact on the human transcriptome from the SETMAR protein methylase, which is a fusion between a SET-domain protein methylase and the *HsMar1* transposase. This work demonstrates that the DNA-binding domain of the transposase targets the enzyme to residual transposon-end sequences, allowing for the regulation of gene expression dependent on methylase activity.

**Figure 1.**
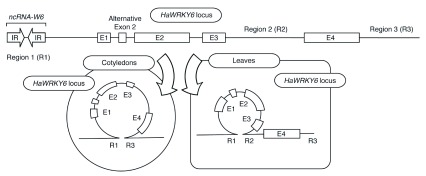
An inverted repeat (IR) transposable element regulates chromatin topology at the *HaWRKY6* locus in sunflower. A simplified representation of the *HaWRKY6* locus is shown, with opposed arrows indicating an IR transposable element and boxed segments indicating exons (E). Regions to which small RNAs (sRNAs) were mapped are indicated (regions R1–R3). The altered chromatin structure of the locus in cotyledons and leaves is diagrammatically presented. The chromatin loop encompasses the *HaWRKY6* gene in cotyledons, enhancing transcription, while an intragenic loop forms in leaves, inhibiting transcription. The locus and exons are not drawn to scale.

Recently, Gagliardi and colleagues ^90^ identified an interesting transposon-based mechanism for the regulation of gene expression at the *HaWRKY6* locus in the sunflower genome. Analysis of expressed sequence tags corresponding to this locus revealed a non-coding RNA derived from an inverted repeat (IR) of the Miniature IR Transposable Element (MITE) family situated 600–800 bp upstream of the *HaWRKY6* transcriptional start site. MITEs are 50-to-500 bp non-autonomous transposable elements with terminal IRs typically found in gene-rich regions of plant genomes ^91^. Transcripts from the IR elements are processed into 24-nucleotide siRNAs, which trigger DNA methylation and nucleate the formation of tissue-specific chromatin loops at the *HaWRKY6* locus ( Figure 1). In sunflower leaves, an intragenic loop forms, comprising the regulatory region of the *HaWRKY6* gene up to its fourth intron. This looped conformation inhibits the expression of *HaWRKY6*, likely by blocking movement of RNA polymerase II. In cotyledons, however, an alternative loop forms, encompassing the full *HaWRKY6* gene and enhancing its transcription. The formation of this loop changes RNA polymerase II directionality, which may reduce transcription of the IR region, decrease siRNA production, and ultimately release the looped conformation. This elegant mechanism highlights the broad, and potentially undiscovered, functions that transposons may fulfill in regulating gene expression.

## Transposon-based phenotypic screening

Transposons have been employed at length as laboratory reagents for the facile construction of mutants, including gene disruption/replacement alleles, promoter fusions resulting in altered timing and levels of transcription, and translational fusions for the construction of various chimeras including epitope/fluorescent protein-tagged products. Relative to chemical treatments for DNA mutagenesis, transposon-based approaches provide a marked and defined mutation that can be easily identified in mutants of interest by virtue of the transposon sequence itself. CRISPR–Cas gene editing is a relevant means of generating genomic mutations. While targeted mutagenesis by CRISPR–Cas is a powerful strategy, CRISPR-based approaches do not readily allow for the scale of mutations needed to saturate a large genome. Furthermore, CRISPR–Cas strategies are not currently applicable for genome-wide screens in multicellular organisms. In contrast to targeted mutagenesis approaches, transposons can be used to generate a larger number of mutations with economy of labor and cost, although transposition bias can complicate genome-wide studies where saturating coverage is desired ^92– 94^. Insertion bias and the gene density of the targeted genome will impact the observed density of coverage by transposon mutagenesis; genomes with relatively small intergenic spaces are more easily saturated by transposon mutagenesis. Strategies employing *in vitro* mutagenesis with subsequent insertion alleles being introduced into the relevant organism by DNA transformation and approaches utilizing transposon mutagenesis *in vivo* have been used for large-scale studies. The applicability of these respective approaches depends on the density of mutations desired and the degree of insertion bias demonstrated by the transposon. *In vitro* transposition systems may provide less insertion bias and greater coverage, although the applicability of such systems is dependent upon the availability of efficient methods for the chromosomal integration of exogenous DNA. Notably, saturating *in vivo* mutagenesis has been performed in yeast ^95^. Many early applications of transposon mutagenesis were used for genome-wide phenotypic analysis in prokaryotes and eukaryotes ^96– 106^, and transposon-based approaches continue to be utilized today.

Recent screening strategies have incorporated barcodes in the transposon, enabling the construction of barcoded mutant libraries that can be effectively multiplexed or analyzed in parallel for large-scale phenotypic analysis ^107^. Helmann *et al.* ^108^ utilized random bar-coded transposon mutagenesis to identify genes contributing to the fitness of the bacterial plant pathogen *Pseudomonas syringae*. In this work, a collection of 281,417 mutant *P. syringae* strains in the B278a background were generated by random mutagenesis using a DNA-barcoded variant of a *Mariner* transposon. The library encompassed 169,826 strains containing an insertion within a known gene, representing 84% of the protein-coding genes in *P. syringae*. By virtue of the incorporated barcodes, amplicon sequencing of the barcoded regions was used as a relative measure of abundance of each mutant strain and a proxy of strain fitness in pooled populations. The analysis identified at least 392 genes predicted to be essential for the growth of strain B278a under standard laboratory conditions. The work further identified a set of *P. syringae* genes required for its colonization of the surface and interior habitats of the bean *Phaseolus vulgaris*, collectively highlighting the utility of barcoded transposon sequencing for genome-wide mutagenesis screens.

Chang and colleagues ^109^ have adapted transposon mutagenesis for genome-wide phenotypic screening in mice through an approach enabling the easy identification of mice with insertions, while requiring relatively modest numbers of mice and researchers. This work utilized a modified form of the DNA transposon *piggyBac* for use in mammalian cells and mammals. Classic systems contain a non-autonomous *piggyBac* transposon cassette, for the delivery of exogenous genes of interest flanked by the *piggyBac* IR sequences, and a transgene expressing the *piggyBac* transposase, enabling induced transposition in the germline ^110^. Binding of the transposase to the IR sequences results in the excision and reintegration of the cassette at another locus. The study by Chang *et al.* ^109^ presents a *piggyBac* construct with a conditionally regulated promoter for gene overexpression and a stop cassette with splice acceptor and poly(A) signal for efficient disruption of target transcription. Furthermore, the transposons are visually trackable, utilizing a red fluorescent protein transgene and a codon-optimized luciferase gene. Luciferase gene activity is disrupted by insertion of the *piggyBac* construct, and luciferase activity is restored upon excision of the insertion, providing a convenient means of visually tracking transposon mobilization. For genome-wide mutagenesis of the mouse germline, a transgenic line was generated carrying 10 copies of the *piggyBac* transposon. By this clever approach, Chang and colleagues implemented a cost-effective and efficient pilot first-generation F1 dominant screen for growth retardation phenotypes in mice.

The *Sleeping Beauty* transposon system has been used for mutagenesis in somatic tissue and holds strong potential utility for the analysis of cancer and other phenotypes both *in vitro* and *in vivo* ^111, 112^. The *Sleeping Beauty* system consists of the eponymous transposase and transposon, initially found in the genome of salmonid fish in the late 1990s ^113^. *Sleeping Beauty* transposons have been used extensively for insertional mutagenesis in embryonic stem cells ^114^, somatic tissues ^115, 116^, and germline tissues ^117– 119^. *Sleeping Beauty* transposons have been used to identify colorectal cancer-related genes in a mouse model ^120^. Recently, Grisard and colleagues ^121^ utilized a *Sleeping Beauty*-based forward genetic screen, coupled with single cell assays, to uncover regulators of metastatic colorectal cancer. The analyses identified the microRNA *MIR23-b* and *BTBD7* as prognostic predictors of colorectal cancer metastasis, illustrating the utility in transposon mutagenesis relative to chemical, radiation, or viral mutagenesis for analyses of putative biomarker functions prior to clinical applications of liquid biopsy assays ^122^.

## Transposons as vectors for gene therapy

DNA transposons have emerged as viable vectors for gene therapy (reviewed in 123), such that numerous proof-of-concept studies of transposons for *ex vivo* and *in vivo* therapy in disease models now exist. Approaches including codon optimization of the transposase, the engineering of hyperactive transposases, and modification of transposon terminal repeats have improved transposition efficacy, enabling stable gene transfer in stem/progenitor cells and in differentiated cell types. With respect to *Sleeping Beauty*, hyperactive variants of its transposase have been generated through methods for *in vitro* evolution and selection ^124^ and structure-based design/molecular engineering ^125^. *Sleeping Beauty* systems have been used for the delivery of transgenes up to 8 kb in length ^126^, and *Sleeping Beauty* has been used in phase I trials to generate CD19-specific Chimeric Antigen Receptor (CAR)-T cells for immunotherapy relevant to the treatment of non-Hodgkin lymphoma and acute lymphoblastic leukemia ^127^. Rational protein design has been used to generate a *Sleeping Beauty* transposase with high solubility and stability that can be effectively delivered with transposon DNA to genetically modify cell lines, embryonic stem cells, hematopoietic stem cells, and induced pluripotent stem cells. This approach has been used to generate CAR-T cells, exhibiting potent antitumor activity *in vitro* and in xenograft mice ^128^. The transposase for *piggyBac* has been modified by approaches utilizing codon optimization ^129^ and the incorporation of site-specific mutations ^130^. The *piggyBac* system is capable of delivering DNA cargo up to 100 kb in length ^131^, including full-length human dystrophin for the treatment of dystrophic mesoangioblasts ^132^. The *Tol2* transposon system can deliver transgenes up to 11 kb in length and has been used in a number of transgenesis studies in zebrafish and other organisms ^133– 135^, although the efficiency of *Tol2*-based gene transfer may not be as high as that observed in *Sleeping Beauty* or *piggyBac* systems ^136^.

## RNA-guided transposon insertion

**Figure 2.**
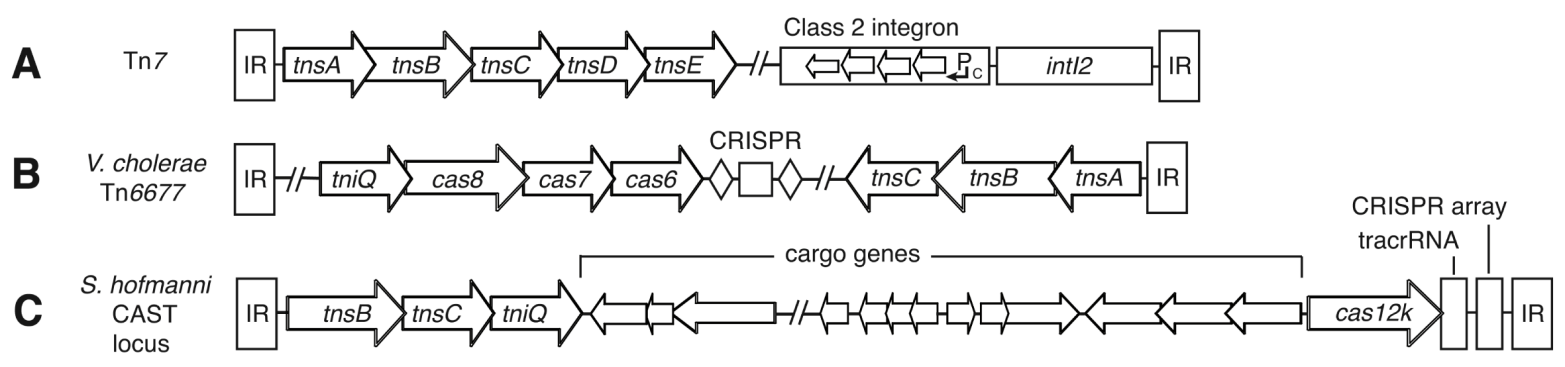
Sequence structure of the Tn7 transposon and derivatives encompassing clustered regularly interspaced palindromic repeats (CRISPR)–Cas-type systems. **A)** The structure of a typical Tn *7* transposon is shown, with terminal inverted repeats (IR) shown as boxes and constituent genes shown as arrows. The diagram is based on the structure of the *Escherichia coli* R721 shufflon. **B)** The structure of the *Vibrio cholerae* Tn *6677* transposon is shown. Tn *7*-derived elements and the CRISPR array are indicated. **C)** A representation of the *Scytonema hofmanni* CAST locus is provided, encompassing Tn *7*-like open reading frames, the effector Cas12k, and the CRISPR array. The loci are not drawn to scale.

RNA-directed transposition provides a promising experimental approach for the generation of targeted insertions, and the CRISPR–Cas system is proving to be important for this work. Bioinformatic analyses have identified CRISPR–Cas systems encoded on some transposons, with the CRISPR-derived sequences potentially fulfilling a role unrelated to host organism defense ^137, 138^. Variants of the *E. coli* transposon Tn *7* ( Figure 2A) have been found to encode CRISPR–Cas systems. Classically, Tn *7* encodes TnsE, which contributes to random Tn *7* transposition in conjugal plasmids and replicating DNA, as opposed to targeted insertion mediated through TnsD at *attTn7* Tn *7* attachment sites ^139^. Tn *7* variants encoding CRISPR–Cas systems lack sequence-encoding orthologs of TsnE, and the CRISPR–Cas systems lack the Cas proteins needed to acquire novel spacers and the nucleolytic activity to cleave targets ^137^. Genes enabling target recognition are still present, suggesting that the transposons might utilize CRISPR effectors, thereby guiding transposition to targets defined by the spacers in the CRISPR arrays.

Two groups have recently demonstrated RNA-directed insertion by Tn *7* relatives containing CRISPR–Cas. Strecker and colleagues ^140^ analyzed a Tn *7*-related CRISPR-associated transposase from the cyanobacterium *Scytonema hofmanni*, called CAST ( Figure 2B). The transposase consists of Tn *7*-like transposase subunits TnsB, TnsC, and TniQ and the type V-K CRISPR effector Cas12k. This Tn *7*-like transposition can be directed to target sites by CRISPR–Cas-mediated RNA-guided targeting. Tn *7* transposition can be reprogrammed to insert DNA into targeted sites in the *E. coli* genome with frequencies of up to 80% without positive selection. Klompe *et al.* ^141^ identified a *Vibrio cholerae* CRISPR–Cas effector complex in the element Tn *6677* ( Figure 2C) that can direct an accompanying Tn *7*-derived transposase to integrate DNA 48–50 bp downstream of a genomic target site complementary to a guide RNA. The Tn *6677* element encodes TnsA, TnsB, TnsC, and TniQ. This transposition involves the formation of a complex between the DNA-targeting complex Cascade and the *cas*-encoded transposition protein TniQ, an ortholog of *E. coli* TnsD. The presence of TnsA allows for cut-and-paste transposition that would result in a simple insertion event. As indicated above, the CAST element lacks TnsA, and thus cleavages at the transposon 5'-ends leading to simple insertions are presumably provided by a host factor and not a component of the transposase. Maximum transposition of the Tn *6677* element occurred with a 775 bp transposon donor, requiring 105 bp of sequence at the Tn *6677*-left terminus and 47 bp of the right-end terminus. Programmable transposition of the *V. cholerae* Tn *6677* element ( Figure 2C) was observed across dozens of unique target sites, indicating the potential utility in these techniques as a means of achieving site-specific DNA insertions in bacteria without the generation of a dangerous double-strand break. Notably, CRISPR–Cas mutagenesis results in a double-strand break for repair by either non-homologous end joining or homologous recombination. RNA-guided transposition holds the potential to enable targeted genomic insertion of transposable elements with potentially large cargoes at selected sites constituting “safe havens”, thereby diminishing the risk of unanticipated insertional mutagenesis.

Aside from the relevance of RNA-guided transposition as a tool for biotechnology, the identification of transposon-encoded CRISPR–Cas variants poses interesting and unanswered evolutionary questions as to the apparent selective advantage in this biological design. CRISPR–Cas-guided transposition is thought to have evolved independently at least three times in Tn *7*-like elements ^137, 138^. As discussed by Dimitriu *et al.* ^142^, this mechanism is far from ubiquitous, suggesting that balanced costs and benefits are at play in the evolution of systems for RNA-guided transposition. The ability of transposons to hijack CRISPR–Cas effector machinery may be advantageous as a means of biasing transposition towards mobile genetic elements for enhanced horizontal transfer; however, it is unclear how these CRISPR–Cas systems lacking genes needed for spacer acquisition would be able to identify rapidly evolving mobile genetic elements. Strecker *et al.* ^140^ suggest that host CRISPR–Cas machinery may capture spacers for insertion into Tn *7*-encoded CRISPR arrays. Klompe and colleagues ^141^ found that the vast majority of type I–F CRISPR–Cas systems within the *Vibrionaceae* family are associated with mobile genetic elements, consistent with the possibility that RNA-guided DNA integration may facilitate the sharing of innate immune systems and virulence mechanisms through horizontal gene transfer. Regardless of the evolutionary pressures that have driven this unexpected interrelationship, RNA-guided transposase systems are primed to be an important and rapidly expanding area of study in biotechnology and evolutionary biology fields.

Along these lines of generating higher efficiency systems for directed DNA delivery, Bhatt and Chalmers ^143^ have recently co-opted Cas9 to target integration *in vitro* through the reconstituted *Mariner*-family transposon *HsMar1*. For this work, a chimeric protein was generated consisting of the *HsMar1* transposase fused to the amino terminus of *E. coli* dCas9. The transposase and Cas9 moieties in the chimera were able to bind their respective substrates. Furthermore, the fusion protein was effective in targeting *HsMar1* activity *in vitro*, resulting in unidirectional integrations with a targeting efficiency of more than 50%. It remains to be determined if this approach will be effective and sufficiently selective *in vivo* in large genomes. Hew *et al.* ^144^ fused a hyperactive form of the *piggyBac* transposase to catalytically dead high-fidelity SpCas9-HF1 (dCas9). The researchers introduced mutations to the native DNA-binding domain of *piggyBac*, decreasing non-specific transposase binding and favoring binding of the chimera by dCas9. By this approach, transposition was directed to the safe harbor *CCR5* sequence using appropriately designed guide RNAs. The insertion profile of *Sleeping Beauty* has been biased through fusion of its transposase, or an N-terminal fragment of its transposase, with DNA-binding and protein dimerization domains ^145, 146^. Ongoing work in the Ivics laboratory is addressing the modification of *Sleeping Beauty* target site selection using dCas9 and a single guide RNA against the *Alu* retrotransposon for integration in genomic regions that are otherwise poor targets for *Sleeping Beauty* transposition ^147^. Cumulatively, the findings speak to the potential utility in harnessing CRISPR–Cas technology for RNA-targeted transposable element integration.

## Transposon-related studies in biological disciplines

The studies above highlight the current breadth of research interests touching on the biology of transposable elements. Mechanisms of transposition and the interplay between transposons and host systems have been foci for intensive research efforts over some time, and the persistent and important questions that remain regarding these topics of study continue to prime investigations of the molecular basis of transposition and its utilization or avoidance of host biological processes. The impact of transposition on gene expression and function at insertion loci is substantial, and the disease implications of transposons as hotspots for mutation are still being understood. Transposons have also been utilized as significant tools for large-scale phenotypic screening, and recent discoveries of CRISPR–Cas-guided transposition hold high potential in facilitating targeted DNA integration without the off-target mutagenic potential of approaches utilizing homologous recombination. Collectively, the coming years are likely to witness expanding interest in transposon biology towards scientific advancements and the establishment of broad human health benefits.
